# Healthcare professionals’ views on implementing the STAR care pathway for people with chronic pain after total knee replacement: A qualitative study

**DOI:** 10.1371/journal.pone.0284406

**Published:** 2023-04-28

**Authors:** Andrew J. Moore, Vikki Wylde, Wendy Bertram, Andrew D. Beswick, Nick Howells, Rachael Gooberman-Hill

**Affiliations:** 1 Musculoskeletal Research Unit, Bristol Medical School, University of Bristol, Bristol, United Kingdom; 2 National Institute for Health Research Bristol Biomedical Research Centre, University Hospitals Bristol and Weston NHS Foundation Trust and the University of Bristol, Bristol, United Kingdom; 3 North Bristol NHS Trust, Bristol, United Kingdom; The University of Sydney, AUSTRALIA

## Abstract

For many people with advanced osteoarthritis, total knee replacement is an effective treatment to relieve pain and improve function. However, 10–34% of people experience chronic postsurgical pain in the months and years after total knee replacement. The Support and Treatment After Replacement (STAR) randomised controlled trial (ISCRTN92545361) evaluated the clinical- and cost-effectiveness of a new multifaceted and personalised care pathway, compared with usual care, for people with pain at three months after total knee replacement. Our objective was to identify factors promoting or inhibiting its implementation, and to inform future training and wider implementation of the pathway. We conducted a prospective process evaluation using qualitative interviews with eight Extended Scope Practitioners and six Principal Investigators from seven trial sites who were involved in delivering the STAR care pathway during the trial. We used Normalization Process Theory as a theoretical framework for qualitative data collection and content analysis. We identified that factors promoting the implementation of the pathway were quick familiarisation with the pathway, valuing patient-centredness, formalising referral processes, and increasing confidence to address neuropathic pain. Challenges to implementation were availability of time and resources, sensitivity in referral process, and ensuring collective understanding of the pathway. These findings have enabled us to make recommendations about the future implementation of the STAR care pathway and will inform the development of a training package, and updated manual for successful delivery in usual care. Furthermore, this model of care has potential value in diverse elective surgeries and pain conditions.

## Introduction

Total knee replacement is a common surgical procedure for treatment of advanced osteoarthritis. Over 100,000 knee replacements were performed annually in the UK’s National Health Service (NHS) before the COVID-19 pandemic [1, 2]. Demand for knee replacement in the UK is projected to increase by over 40% by the year 2060 [3]. These rises are global: for instance, in the USA over 1 million people were predicted to receive a total knee replacement in 2020 [4]. While most patients benefit from knee replacement, 10–34% experience chronic post-surgical pain—pain that develops or increases in intensity after surgery and persists at least 3 afterwards [5, 6]. For instance, in a UK study, among 258,386 people with total or unicompartmental knee replacement, 16.9% reported chronic pain likely to affect their quality of life negatively at six months after their operation [7]. Some people with chronic pain after knee replacement experience considerable distress and report feeling abandoned by healthcare [8] or believe that nothing further can be done for their pain [9]. Prevention of the development of chronic pain among individuals who might be at higher risk has been suggested as a good approach, but such identification is known to be challenging [10]. This means that a focus on pain after surgery remains a key point for intervention, including because there has been no clear referral and treatment pathways in place for such individuals [11, 12].

The STAR care pathway is a multifaceted personalised care pathway for people with troublesome pain at three months after total knee replacement [13]. The pathway was designed in line with UK Medical Research Council guidance on complex intervention development [14].

The pathway comprises an assessment clinic appointment with a trained Extended Scope Practitioner (ESP: an allied health-care professional with specialist orthopaedic training), referral to existing services for treatment, and up to 6 follow-up telephone calls over 12 months. ESPs who delivered the pathway received specialised training in the assessment of patients with problematic knee replacements, that included a detailed intervention manual and ongoing support from the research team. The 1-hour assessment aims to identify potential underlying causes for chronic pain and enable timely onward referrals to services for treatment. The clinical and cost-effectiveness of the STAR care pathway was evaluated in a multi-centre randomised controlled trial [15]. Results of the trial were published in full in 2022: the pathway was found to be clinically effective and cost-effective for reducing pain severity and interference over a 1-year period for people with pain at 3 months after total knee replacement surgery [16].

As highlighted by David Beard in his commentary on the STAR trial, the design of the trial was a pragmatic, pathway-based design rather than a direct comparison of a highly specific intervention, and “one that usefully accounts for real-world variation in treatment content and fidelity” [17]. We therefore report here on the experiences of real-world delivery of the STAR care pathway within NHS hospitals during the published trial [16]. We suggest that understanding the implementation potential of the pathway provides information for healthcare providers that may wish to consider putting the intervention into practice. Specifically, we describe qualitative research about the experiences of the healthcare professionals who delivered the pathway during the trial. More broadly, understanding implementation of new interventions and how they become embedded into practice provides understanding the success or failure of interventions, and the factors that shape intervention outcomes [18].

This study sought to understand healthcare professionals’ experiences of putting the STAR care pathway into practice in a multi-centre randomised controlled trial. This aimed to identify and describe barriers or facilitators to implementation and to produce recommendations for any delivery of the pathway should providers wish to do so. These recommendations will inform future development of a training package, updated manual and recommendations for delivery in usual care.

## Methods

We used qualitative methods to explore and characterise the experiences of healthcare professionals’ implementation of the STAR care pathway. Qualitative methods embedded in clinical trials play an important role in understanding experiences of implementation [18]. The study received University of Bristol Faculty of Health Sciences Research Ethics Committee approval (ref. 83162) on 1st May 2019 and HRA approval on 1st June 2019 (ref. 19/HRA/3168). Our reporting of research methods includes items of relevance to the study design and is in keeping with SRQR guidelines [19].

### Participants, recruitment and sampling

The study took part in NHS hospitals in North, Central and South England, and Wales. Extended Scope Practitioners (ESPs) and Principal Investigators (PIs) from each of the eight trial sites were invited to participate in this embedded process evaluation. All were individuals who were involved in delivering the STAR care pathway in the trial.

ESPs and PIs were known to the team as they were in regular contact about the trial. The nature and aims of the research meant that the participants were all involved in the delivery of the STAR care pathway in the trial. Three of the PIs were also collaborators on the STAR Programme Grant application and had advised on development of the care pathway. Their inclusion as participants is appropriate as they have a practical knowledge and experience of how the STAR care pathway was implemented. In any research programme those involved in developing it may also be those who go on to deliver the research. We have ensured that their views are balanced with those of the three PIs who were not previously involved in the STAR Programme.

Potential participants were invited to take part in this qualitative study after sites closed to trial recruitment in May 2019. The timing of the invitation was to ensure that included ESPs and PIs had experience of implementing the STAR care pathway. Interviews were conducted between August and October 2019. The qualitative study lead (AJM) sent ESPs and PIs an information pack via email describing the purpose of the study and what was involved. Potential participants responded by reply to the email and a mutually convenient time for telephone interview was arranged. Participants completed an electronic consent form before interview, including consent to audio-recording and publication of anonymous quotations.

Our approach to sample identification and sample size was pragmatic and aimed to achieve inclusion of participants from all eight trial sites. All clinicians with experience of implementing the STAR care pathway were invited to take part. In total, fourteen ESPs and PIs from across seven sites participated in the study out of a potential 21 ESPs and PIs from eight sites.

### Data collection

Data collection comprised semi-structured telephone interviews. We used Normalization Process Theory (NPT) to inform design of the data collection materials and subsequent analysis. NPT provides a theoretical framework to explore and explain the work involved in implementing new complex interventions into routine practice [20] and can be used to capture information needed to enable interventions to become embedded and sustained within organisational settings [21]. Within NPT, the work of implementation can be conceptualised through four core constructs that represent the different kinds of work that people do when implementing a new practice:

- Coherence: how people individually and collectively make sense of a new intervention
- Cognitive Participation: how people build a community of practice around a new intervention
- Collective Action: the operational work that people do to enact a new intervention
- Reflexive Monitoring: the appraisal work that people do to understand how a new intervention affects them [20, 22].

These four core constructs form the basis of the Normalization MeAsure Development (NoMAD) instrument, which assesses and monitors normalisation processes from the healthcare professionals’ perspective. NoMAD consists of three general items to assess familiarity with the intervention and whether the intervention is a normal part of the healthcare professional’s work or could become a normal part of their work; and 20 items that represent the four core constructs of NPT [21]. NoMAD has been used to identify, understand and assess implementation processes from the perspective of those directly involved in implementing interventions in various areas of healthcare. Examples include its use in relation to weight management [23], primary care services for transgender individuals [24], surgical safety checklists [25] and cognitive behavioural therapy [26]. We previously used NoMAD to assess the views of healthcare professionals who were involved in development of the STAR care pathway before its delivery in the trial [13]. In our previous publication we highlighted that intervention development needs to be flexible and adapted to context and setting. We also acknowledged the potential for complementary data collection alongside NoMAD, for instance use of telephone interviews to provide further depth [13]. We addressed those considerations in the design of the current study by using the NoMAD tool as the basis for an interview topic guide.

The interview topic guide was structured using the NoMAD questionnaire items [see S1 Appendix]. Participants were asked about their familiarity with the intervention; views about the potential of the intervention to become a normal part of healthcare professional’s work; and thoughts about coherence, cognitive participation, collective action and reflexive monitoring. Interviews were conducted by the lead author AJM (male, PhD, Senior Lecturer in Musculoskeletal Health Services Research) with a background in health sociology and significant qualitative research experience.

### Analysis

Audio-recordings were transcribed, anonymised and uploaded to NVivo11 data management software [27]. We used the NoMAD items as a framework to guide content analysis [28]. Extracts from the interview transcripts were first coded inductively. Codes that shared similarities were then grouped and deductively assigned to themes according to the three global assessment questions, and the four core constructs of NPT. The content of these five themes was then explored with a focus on participants’ experiences of implementing the STAR care pathway and any barriers and facilitators to implementation. To provide rigour in the coding process, four transcripts were independently coded inductively and then codes were agreed and applied to the full dataset. Double coding was conducted by two team members with different disciplinary perspectives: RGH (Professor of Health and Anthropology) and WB (Trial Manager).

## Results

Eleven PIs, and 10 ESPs from across the eight trial sites were invited to participate. Eight ESPs and six PIs from seven trial sites participated. All PIs were consultant knee surgeons and are referred to as consultants from hereon. All consultants were male. All ESPs were experienced musculoskeletal physiotherapists and included 4 males and 4 females. Participants had been in their role on the STAR project for between 18 months and 4 years at time of interview. The sites were located in North, Central and South England, and Wales. We have not provided a table of participant characteristics to protect anonymity.

We present the findings using the three general items from NoMAD, which aimed to elicit a global sense of the potential for normalization, and then the four core constructs from NPT. Subheadings describe the main implementation considerations apparent within each construct. Illustrative quotations relating to participants’ experiences are in S1–S5 Tables.

### A global sense of the potential for normalisation of the STAR pathway (S1 Table)

ESP participants quickly became familiar with the care pathway as they delivered it. They found that the intervention manual was useful, with one ESP reflecting that they still used it as a prompt. ESPs reported that some elements of the pathway had already been part of their clinical role before the trial, including follow-up and referral for pain. However, there were other elements that were new to their practice, including regular follow-up telephone calls, a raised awareness of the potential for complications and the assessment of neuropathic pain. Participants felt there was the potential for the STAR pathway to become a normal part of practice, if sufficient time and resource was available and if the trial findings indicated that the pathway was beneficial compared with usual care. The results of the trial had not yet been released at the time of the interviews.

### Coherence: How did healthcare professionals individually and collectively make sense of STAR? (S2 Table)

Participants identified areas in which the care pathway differed from their usual practice.

#### The duration of the clinic allows more focus on the patient

The clinic was much longer at 60 minutes than standard 15–20 minute follow-up appointments, and participants felt that patients benefited from having more time to talk and reflect on different aspects of their surgery and on problems they had experienced in their recovery. Likewise, the participants appreciated having more time to read patients’ notes and to formulate treatment plans.

#### Follow-up telephone calls provide an opportunity for support, but require adequate resources

Another difference from usual care, was the follow-up telephone calls for patients on the STAR care pathway. ESPs often acknowledged the requirement for extra resources to provide these. One ESP felt reassured however to be providing a link for patients as they felt that often those with problematic knees had experienced communication difficulties in the system somewhere, and the follow-up calls provided an extra opportunity to support those patients. Another ESP suggested that the follow-up telephone calls would be a better use of clinicians’ and patients’ time, being more convenient for the patient and possibly less expensive than face-to-face reviews in usual care.

#### STAR is seen as a positive, proactive approach to provision of support for patients at risk

Another key difference between the STAR care pathway and usual care is that the pathway demands a more proactive approach to identifying patients at risk of developing ongoing pain. Intervening or as one participant suggested ‘catching’ patients in need of extra care in the early stages of recovery was seen as a positive strategy.

#### Consultants needed to be made aware of the purpose and process of the STAR care pathway

Overall, there was generally a shared understanding of the purpose of the care pathway among those directly involved in its delivery. However, ESPs in four centres reported that some surgeons were concerned when their patients were seen in the STAR clinic or referred back to them, and they suggested that this was possibly because the surgeons perceived their work was being checked by the ESPs, or that something had gone wrong with the knee replacement. ESPs at two sites suggested they were not sure how well some surgeons understood the reasoning behind referrals back to them, or how the STAR care pathway worked. A consultant surgeon suggested that it was often “a surprise” when a patient was referred back to them because they had expected the patient to be doing well, especially if they were recovering well at the 6-week follow-up. One ESP also suggested that the STAR care pathway gave them more confidence to justify their decisions about referring on for neuropathic pain medication.

#### Healthcare professionals valued the focus on neuropathic pain contained in the STAR care pathway

For the healthcare professionals who delivered it, the care pathway changed the nature of their work. They valued having protected time to address psychosocial factors and neuropathic pain, which previously many had not had the training or time to focus on, even though they suspected neuropathic pain was the cause of some patients’ poor outcomes.

### Cognitive participation: How did healthcare professionals build a community of practice to support the STAR care pathway? (S3 Table)

#### Enthusiastic, experienced ESPs are needed to deliver the STAR care pathway and orthopaedic consultants need to provide leadership and motivation

In terms of building a community of practice to support the implementation of the STAR care pathway a number of key individuals were seen as essential to driving the intervention forward: ESPs thought the research and administrative staff were key to organising clinics. One consultant noted that while research staff were key in the trial, if the care pathway was implemented more widely in the future then it would likely need to be enthusiastic and experienced ESPs who would drive the intervention delivery. If this took place then consultants would take responsibility for leadership and motivation of the team to continue pathway delivery.

#### ESPs and orthopaedic consultants feel that the STAR care pathway is a legitimate part of their role

Participants felt that delivery of the care pathway was a legitimate part of their role, particularly where local pathways were already similar to STAR. All participants were open to working in new ways to deliver the pathway and would continue to support it, if it were to be implemented into usual care. One consultant reported that they felt the pathway was aligned with a general shift in focus towards listening to patients more after joint replacement.

### Collective action: What operational work did healthcare professionals undertake to deliver the STAR care pathway? (S4 Table)

#### The STAR care pathway can be integrated but requires extra resources to do so

In terms of collective action–the operational work that people did to ensure the STAR care pathway was delivered–participants were positive about integrating the pathway into their existing work. They thought that many aspects of the pathway were similar to usual care in terms of the clinical skills required. However, the 1-hour clinic and follow-up telephone calls required more time than usual care workload allocations, and so would need funding and support from management. They felt that if the trial showed that the pathway was of benefit then this could be provided.

#### The STAR care pathway can have a positive effect on working relationships and communication

In terms of the care pathway’s effect on working relationships, as stated in the theme addressing coherence, some ESP participants reported that they were unsure how surgeons might feel about patients being referred back to them, and felt that such referrals should be managed, carefully and sensitively.

A positive effect on working relationships was noted by one consultant who reported how the pathway had contributed to the creation of a team of expert clinicians, highly specialised in knee replacement and follow-up, and that this benefitted patients. Another consultant felt that STAR had improved communication of information across their clinical team, with regard to patients who were struggling. Training and support for delivery of the STAR care pathway was felt to be excellent, and ESPs reported feeling confident that they were supported by the trial team.

### Reflexive monitoring: How do healthcare professionals reflect on how the STAR care pathway affects them? (S5 Table)

In terms of reflexive monitoring, participants reflected on whether they felt the STAR care was worthwhile for them, and how it affected their own work.

#### The STAR care pathway increased consultants’ and ESPs’ awareness of the nature of pain, and allowed them to explore patients’ beliefs about joint surgery

One consultant reflected on how they had become “more careful about the nature of pain” because of the pathway, and that their threshold had become lower for identifying and treating psychosocial issues and neuropathic pain. Another ESP valued the focus on neuropathic pain and reported that their experience of delivering the pathway confirmed their suspicion that some patients after knee replacement were susceptible to development of neuropathic pain. Others found that the STAR pathway provided validation of their existing local pathway and suggested that the STAR pathway allowed them to focus more on exploring aspects of patients’ beliefs about joint replacement surgery. They felt that this reflected a shift in focus towards biopsychosocial approaches and expectation management.

#### Suggested modifications to the pathway

In terms of whether healthcare professionals would modify how they worked with the STAR care pathway and follow-up telephone calls, two ESPs explained that when patients did not answer the calls, this could be time consuming. One ESP suggested that a future modification could include asking patients to call and book a 10 minute-telephone consultation with a physiotherapist rather than asking the professional to make contact with a patient in the first instance.

## Discussion

We evaluated implementation of the STAR care pathway for chronic pain after knee replacement. The study elicited and explored views of the healthcare professionals involved in delivery of the care pathway in a randomised controlled trial. Allen and colleagues have emphasised the importance of incorporating conceptual frameworks to guide implementation in trials to enhance the translation of findings [29]. We used NPT, operationalised through NoMAD to frame collection of qualitative data. Our descriptive content analysis identified participants’ responses that mapped onto the four core constructs of NPT.

Our findings indicate that healthcare professionals quickly became familiar with the STAR care pathway and felt it had potential to become a normal part of their practice. ESPs were already familiar with many elements of the pathway in their current clinical roles. However, the pathway differed from normal practice in many respects: that the duration of the clinic provided patients with more opportunity to reflect on their experience of surgery and recovery; the pathway gave increased focus on identification of potential complications including neuropathic pain; and the pathway provided regular telephone follow-up. Healthcare professionals felt that decisions by healthcare providers about future implementation would depend on trial results. The results of the trial have now been published and indicate that the STAR care pathway is both clinically beneficial and cost-effective over a 1-year period for patients with chronic pain at 3 months after total knee replacement [16].

Our evaluation of the implementation potential of the STAR care pathway indicates that healthcare professionals valued the patient-centredness of the intervention. Training during the trial enabled and empowered ESPs to identify and provide support for patients at-risk of developing chronic post-surgical pain. During the trial, those with problematic knee replacements, were referred on to appropriate services. ESPs also valued the additional focus on neuropathic pain, which some had previously suspected was a cause of chronic post-surgical pain. This is supported by the baseline information about trial participants, which show that more than half reported neuropathic pain characteristics [30]. Previous research has shown that clinicians often struggle to help patients with chronic post-surgical pain because of a lack of clear guidance and referral pathways [11]. The STAR care pathway provides clear guidance on referral pathways and has formalised referral processes enabling ESPs to refer patients to appropriate services. We found that, across all seven included sites, healthcare professionals were open to working in new ways with the STAR care pathway and would continue to support it.

Potential challenges to implementation include the extra resources needed for the clinic and telephone follow-up, which required more time to deliver. Healthcare professionals felt there may be need for further funding and support from management. Some ESPs also felt that the telephone follow-ups may save time and costs compared with face-to-face consultations. In a separate interview study with patients who took part in the STAR trial, we found that patients derived reassurance and encouragement from the clinic and telephone calls [31].

A key aspect of implementation is the need for a collective understanding and awareness of the intervention. We found that in some centres, surgeons who were not directly involved in the trial did not know why patients whom they had treated were receiving assessment in a STAR clinic and who were sometimes referred back to them. Some were surprised or disappointed to see their patients in STAR having thought they were recovering well at the six-week post operative clinic appointment. We suggest that when implementing the STAR care pathway, all clinicians who provide support for patients with knee replacements should be made aware of and be engaged with the pathway and any changes in referral processes.

Our main theoretical contribution has been to extend the use of NPT to the implementation of a clinical care pathway for chronic post-surgical pain after knee replacement. Our findings complement those of a previous UK study, which shows that when successfully implemented, care pathways encourage a systematic approach that can improve referral and access to appropriate care, promoting person-centred care, better multidisciplinary communication, and management of at-risk patients [32]. In our study, training and support from the trial team, and the inclusion of orthopaedic consultants as PIs providing local leadership and management, ensured that ESP were well supported to deliver the pathway. Previous studies have shown that care pathway implementation can fail in sites where there is insufficient training or strong leadership [33]. We also found that implementation could be challenging if there is a lack of collective understanding. The core concept of coherence has been shown to be especially important where implementation can fail if there is a lack of collective understand about the purpose of the intervention amongst those delivering the intervention [34, 35]. We find this also extends to those involved in supporting patients receiving the intervention.

### Strengths and limitations

Study participants were from 7 out of 8 trial sites. The inclusion of an eighth site might have produced additional findings, however, the sample size of 14 participants from seven sites across England and Wales provided a rich data with reasonable geographic variation across the NHS, although the absence of study sites in Scotland and Northern Ireland may have impacted on future transferability if NHS systems contain features that make a difference to implementation. Healthcare professionals comprised including consultants and ESPs, which was appropriate given the care pathway’s design and delivery. In terms of data collection and analysis, use of NoMAD as a topic framework helped participants to identify and explore in detail issues relevant to implementation. A deductive content analysis provided a way to examine the core constructs of NPT that underpin NoMAD. This is also the first study that we are aware of that has used the NoMAD instrument as a topic framework for interviews to explore in detail issues relevant to implementation.

As previously described, some of the participants were known to the team as they were in regular contact with team members about the delivery of the trial. The interviewer had met three participants previously in a training session provided to the participants. Although this could be construed as a potential limiting factor, as social scientists we remained aware of the relationship, and on reflection feel that this was not detrimental to data collection.

## Conclusion

Our findings suggest that the STAR Care Pathway is supported by healthcare professionals who delivered it, and that it has the potential to be implemented successfully into practice. At the time of writing, the STAR care pathway has been adopted into usual care at one NHS hospital, and a training package is in development. Healthcare professionals valued the patient-centred nature of the care pathway and its use in identification of the nature of chronic post-surgical pain. Extended Scope Practitioners delivering the pathway felt it enabled them to refer patients to appropriate services. Wider learning from this study includes the importance of a collective understanding to support the successful implementation of an intervention. Implementation of an intervention is more likely to be successful if healthcare professionals—who may not be delivering the intervention but who support affected patients—are aware of and engaged with the intervention.

## Supporting information

S1 Appendix STAR implementation interviews topic guide. (PDF)

S1 Table Illustrative quotes indicating the normalization potential of STAR. (PDF)

S2 Table Illustrative quotes indicating coherence. (PDF)

S3 Table Illustrative quotes indicating cognitive participation. (PDF)

S4 Table Illustrative quotes indicating collective action. (PDF)

S5 Table Illustrative quotes indicating reflexive monitoring. (PDF)
