# Supplementary material for: Healthcare professionals’ views on implementing the STAR care pathway for people with chronic pain after total knee replacement: A qualitative study
Source: PLoS One. 2023 Apr 28;18(4):e0284406. doi: 10.1371/journal.pone.0284406 (PMC10146502; doi:10.1371/journal.pone.0284406)
Supplement: S1 Appendix — (PDF) [file pone.0284406.s001.pdf]

STAR Implementation Interviews Topic Guide

Introduction: Discuss how the interview will be recorded, issues of confidentiality, anonymisation and the aim of the research.

"The aim of this interview is to understand your experiences of implementing the STAR care pathway, its strengths and weaknesses and how these can possibly be overcome. We'll complete the NoMAD questionnaire as part of the interview and use this to structure the discussion, so as you answer each question I'll ask you about your answers. Not all of the questions might apply directly to you if you haven't been directly delivering the intervention, but we can still discuss your thoughts about these."

Researcher to request consent from the participant and to check that the participant is happy to take part in the interview and to be recorded.

"Do you have any questions at this point or are you happy to start?"

1. How would you describe your professional role in relation to STAR? (e.g. Extended Scope Practitioner, Principal Investigator)

2. For how many years have you worked in this role?

3. When you deliver a STAR assessment clinic or follow-up telephone call, **how familiar** does it feel to you?

4. Do you feel that the STAR assessment clinic and follow-up calls **are currently** a normal part of your work?

5. Do you feel the STAR assessment clinic and follow-up **will become** a normal part of your work at your hospital?

6. Do you feel that the STAR assessment clinic, and follow-up calls differ from your usual ways of working? *If so, how?*

7. Do you believe that staff in your organisation have a shared understanding of the purpose of the STAR assessment clinic and follow-up calls?

8. Do you understand how the STAR assessment clinic and follow-up calls affect the nature of your own work? *How does it affect the nature of you work?*

9. Can you see the potential value of the STAR assessment clinic and follow-up calls for your own work? *How?*

10. Are there key people who drive the STAR assessment clinics and follow-up calls forward and who get others involved? *Who are they?*

11. Do you believe that participating in the STAR assessment clinic and follow-up calls is a legitimate part of your role?

12. Are you open to working with colleagues in new ways to use the STAR assessment clinics and follow-up calls?

13. Will you continue to support the development of the STAR assessment clinic and follow-up calls?

14. How easily can you integrate the STAR assessment clinic and follow-up calls into your existing work? *What helps or hinders integration?*

15. Does the STAR assessment clinic and follow-up calls disrupt working relationships?

16. Do you have confidence in other people's ability to conduct the STAR assessment clinic and follow-up telephone calls?

17. Is work assigned to those with the appropriate skills to conduct the STAR assessment clinic and follow-up calls?

18. Is sufficient training provided to enable staff to implement the STAR assessment clinic and follow-up calls?

19. Are sufficient resources available to support the STAR assessment clinic and follow-up calls?

20. Does management adequately support the STAR assessment clinic and follow-up calls?

21. Are you aware of reports about the effects of the STAR assessment clinic and follow-up calls?

22. Do your colleagues agree that the STAR assessment clinic and follow-up calls are worthwhile?

23. Do you value the effects that the STAR assessment clinic and follow-up calls have had on your own work? *What sort of effects have they had, if any?*

24. Do you think that feedback about the STAR assessment clinic and follow-up telephone calls can be used to improve it in the future?

25. Can you modify how you work with the STAR assessment clinic and follow-up calls? *Are there ways in which you feel the STAR assessment clinic could be improved?*

26. Do you have any further comments about the STAR trial?

- Are there other things that you would like to tell me?
- Thank you for taking part
- We would like to send you a summary report of the study's findings, would you like to receive this from us?

END
