# Supplementary material for: Healthcare professionals’ views on implementing the STAR care pathway for people with chronic pain after total knee replacement: A qualitative study
Source: PLoS One. 2023 Apr 28;18(4):e0284406. doi: 10.1371/journal.pone.0284406 (PMC10146502; doi:10.1371/journal.pone.0284406)
Supplement: S1 Table — (PDF) [file pone.0284406.s002.pdf]

S2 Table – Illustrative quotes indicating the normalization potential of STAR

Participant identifiers correspond to site and either Extended Scope Practitioner (ESP), or Consultant (COS).

"Yeah, quite familiar now, cos I've done enough of them that it's quite a fairly routine process, if that's what you mean? I mean what the assessment clinics – it's very similar to what I would do clinically anyway in my role. It's just a bit more formalised." (Site 1/ ESP2)

"[*How long did it sort of take you to get familiar with it?*] I suppose erm probably maybe three patients to sort of really get you know, a good flow." (Site 4/ESP1)

"I mean the manual was really good that they produced at the beginning. You know, what they wanted you to ask the patients and things. I think that was good." (Site 1/ESP1)

"More familiar now. Initially I used the guidance book to help me, and I still use that as a prompt." (Site 5/ESP1)

"We don't do any telephone follow-ups or consultations. So, it's just particular to STAR really. And I feel quite used to doing the phone calls now within the STAR trial, but it's not something that we've taken on to do as part of our normal role with – in the knee service." (Site 1/ESP2)

[*Are there any new aspects of it? Things that you weren't used to doing?*] Yeah, I think so. I think STAR has been good for me because it's erm improved my knowledge of chronic pain and the management of it in the early stages. You know, simple management like referral for anti-neuropathic pain drugs that we ask the GPs to consider, just reminding me about things like CRPS […] It's raised my awareness of some of the potential complications […] I think it's just raised my awareness of more the pain side of it." (Site 1/ESP2)

"We see people back in clinic if we deem appropriate. Occasionally, I make the odd phone call, but generally I wouldn't phone them up as I would do with the STAR clinic. I would see them back in clinic. If I felt there was erm a clinical issue or need, I would see them back […] So if I felt there was a pain issue then they'd be off to obviously the pain management, physio or hydrotherapy etcetera. So, from that point of view, it's similar to STAR." (Site 2/ESP1)

"I think it depends on what the study shows, it depends on whether it shows any difference or not." (Site 1/COS1)

"[*Do you think it could become a normal part of work at the hospital?*] I think it could be yes. It's just in its current format would be you know, would be difficult within time constraints at the moment." (Site 5/COS1)

"I Just don't think it – you know, unless they're willing to fund erm you know, additional time. If there was obviously a cost benefit from actually doing it and I suppose the study's research will help inform that. Ern, my honest answer is I don't think that it would be – yeah, I just don't think that would be – there isn't just the manpower I think to do it." (Site 2/ESP1)
