# Supplementary material for: Healthcare professionals’ views on implementing the STAR care pathway for people with chronic pain after total knee replacement: A qualitative study
Source: PLoS One. 2023 Apr 28;18(4):e0284406. doi: 10.1371/journal.pone.0284406 (PMC10146502; doi:10.1371/journal.pone.0284406)
Supplement: S2 Table — (PDF) [file pone.0284406.s003.pdf]

**S3 Table – Illustrative quotes indicating Coherence - how people individually and collectively make sense of STAR**<br><br>**Participant identifiers correspond to site and either Extended Scope Practitioner (ESP), or Consultant (COS).**
"I think for many patients that are struggling with pain after knee replacement, they get an awful lot out of an opportunity to simply talk it through with somebody." (Site 1/COS1)<br><br>"I think a really important part of this has been the fact that you know, someone's listening. Someone's interested, someone that knows what they're talking about and takes time to listen to a patient and see them and explain things. I think that's been a huge bonus for patients in this." (Site 5/COS1)<br><br>"I think they value the chance to tell you what's happened to them, that's all part of the process and they need adequate time I think to talk about the surgery and how they've recovered and what problems they've had along the way and I think that sort of possible slightly cathartic thing is quite good. So, it's just having someone to listen to it." (Site 1/ESP2)<br><br>"You have 15 minutes [in standard care] to read the notes, see the patient, come up with a plan, write – dictate your plan and it's just not very long. Especially if people have complex problems." (Site 2/COS2)<br><br>"I think obviously there's more commitment to following people up on the telephone than we would normally do. A lot of the times it was reassuring to me to see that kind of the standard processes were in place in terms of if the patient is having problems, there was already follow-up in place for the consultants or with physio. So it was kind of acting as a link person. But it was reassuring to see that the system was helping. Often the problem patients are the people that weren't satisfied; there was an issue with communication somewhere along the line. So I think having the [telephone] calls and having an opportunity to kind of help with that and having some extra time could help that." (Site 5/ESP1)<br><br>"We tended to do more face-to-face clinic appointments and then once we were happy with that move patients to a virtual. Whereas STAR involved kind of some follow-up phone calls, which arguably is probably a better use of time […] and probably cheaper to deliver too. [Can you say why they're better or why you feel they might be?] I think what patients want a lot of time is a convenient contact rather than having to sit and wait around." (Site 2/COS1)<br><br>"I think currently the mechanism would be that you almost rely on patients contacting you or through the GP … as opposed to this proactive approach of trying to predict who might get pain and catch them at an early stage and check how pain is, whether it's improving or progressing by the time of intervention." (Site 5/COS1)<br><br>"And a good thing is that most people do improve but there's few that don't by being able to identify and hopefully intervene on those earlier hopefully we're helping them." (Site 1/COS1)<br><br>"Some of them possibly think that we're looking to see whether there's something wrong from an orthopaedic point of view which then concerns them and puts them off a bit" (Site 4/ESP1).<br><br>"One consultant got a little bit upset because obviously we were referring [patient] back for an opinion … the patient went back and relayed the information, probably not in the best way and then obviously it upset the consultant" (Site 6/ESP1).

“They’re not anti it at all, but all I’m saying they view it as a thing of thinking ‘Oh dear, that must mean I must have a problem with my knee replacement’.” (Site 2/ESP2)

“So the research team, had a good understanding of it, cos they had to have. The orthopaedic surgeon in charge of the patient, I’m not always sure that they were particularly clear or necessarily interested with what was going on […] Just cos they weren’t directly involved in the trial probably […] They have to be very selective as to what they get involved with.” (Site 3/ESP1)

“I was never quite sure how on-board all the consultants were with it. You know, if I would write back to them, you’re making sure you kind of do that in a sensitive and professional way, sort of if they would review someone who perhaps I wouldn’t normally send back to the consultant for a review, because it’s STAR protocol you know, we’d send them back to be reviewed by the consultant. You know, wonder if they understood the reasoning behind that.” (Site 5/ESP1)

“A lot of the time it’s like ‘Oh, I’m surprised that erm, that patient’ or ‘Oh, that’s interesting’. Yeah, it’s often erm - you know, maybe you get a name that you’re surprised that erm ‘Oh I thought they might be doing better than that at this stage’ or you know. Yeah, a little bit of disappointment sometimes if you get [laughs] – yeah […] often you’ve already seen them at the six week stage, this is why it’s more surprising, you’ve often seen them at six weeks and thought to yourself ‘Yeah, they were doing fine. There was nothing of concern there’. And the obviously the recruitment and the intervention is happening a few weeks on from that.” (Site 5/COS1)

“I think I had one consultant write back and say, ‘Well why have you sent this patient back for neuropathic pain medication?’ […] And I just wrote back and said ‘Well this is the guidelines, this is what’s been said’ you know, ‘It’s a low threshold of referral and these are the reasons why’. So, it gave me – to us it gives you the confidence then to say to someone ‘Well this is the reason why’.” (Site 6/ESP1)

“It’s been interesting sort of looking at the pain scores, the depression and anxiety, because I certainly wouldn’t look at that in the past … But sort of thinking about neuropathic pain and CRPS [chronic regional pain syndrome] made me think a bit more about that going forwards … I guess with the HADS scores you know, the anxiety and depression ones, we probably don’t have time in clinic to do that ourselves.” (Site 1/ESP1)

I think STAR has been good for me because it’s erm improved my knowledge of chronic pain and the management of it in the early stages […] Obviously we’re always looking out for various complications when we see people post-knee replacement, but it just – it kind of formalised things, cemented things a bit more in my mind. So, I feel that my management of patients following knee replacement has improve. It’s raised my awareness of erm – yeah, some of the potential complications.” (Site 1/ESP2)

“The really interesting thing – and we kind of had an idea that this was the case before, but maybe not to the extent, but how TKRs appear to present with neuropathic pain afterwards. And how the outcome really can be not very good.” (Site 3/ESP1)
