# Supplementary material for: Healthcare professionals’ views on implementing the STAR care pathway for people with chronic pain after total knee replacement: A qualitative study
Source: PLoS One. 2023 Apr 28;18(4):e0284406. doi: 10.1371/journal.pone.0284406 (PMC10146502; doi:10.1371/journal.pone.0284406)
Supplement: S3 Table — (PDF) [file pone.0284406.s004.pdf]

| **S4 Table – Illustrative quotes indicating Cognitive Participation - how people build a community of practice around the STAR pathway**<br><br>**Participant identifiers correspond to site and either Extended Scope Practitioner (ESP), or Consultant (COS).** |
|---|
| "The research nurse has been brilliant and has organised things really well and sort of made my part of it a lot easier." (Site 3/ESP1)<br><br>"It is labour intensive in terms of coordinating all of that … certainly the admin side of things and the research coordinators have been absolutely key […] She's organised the appointment times and prepped the patients. So that was invaluable really" (Site 5/ESP1)<br><br>"Having an ESP that is enthusiastic about it and keen to continue to deliver it and deliver it as well as they possibly can and make a success of it is the most important of all." (Site 1/COS1)<br><br>"So you need – you need key experienced individuals in the roles that are undertaking the service provision for looking after the patient's painful or problematic knee replacements. But at the same time, it needs to be – the message needs to have strong leadership locally and generally that comes from – well certainly initially it comes from sort of consultants down […] to begin with it really needs a lot of certainly sort of senior input to coordinate and motivate the team to carry it on. (Site 2/COS2)<br><br>"Absolutely […] we ought to be focusing on those that don't do so well, why they don't do so well and how we can help them do better. So yeah, that's very much a big push for me." (Site 2/COS1)<br><br>"Yeah, yeah. I think if it broadens your horizons to managing patients, then yeah, it's got to be. It can only be a positive really". (Site 6/ESP1)<br><br>"My background is as a physiotherapist, but I don't actually carry out any physiotherapy work in my orthopaedic capacity here. I sign post people to physiotherapy, hydrotherapy, to pain management or for further imaging or back to see the consultant if I feel that there's any sort of issues […] so from that point of view, I suppose it is similar to STAR or STAR's similar to the normal clinic." (Site 2/ESP1)<br><br>"Yeah, absolutely, yes. I think in knee replacement now a lot of the focus has become on the importance of good discussion and decision making before even contemplating the surgery and then important discussion and, listening to patients afterwards as well." (Site 5/COS1) |
