# Supplementary material for: Healthcare professionals’ views on implementing the STAR care pathway for people with chronic pain after total knee replacement: A qualitative study
Source: PLoS One. 2023 Apr 28;18(4):e0284406. doi: 10.1371/journal.pone.0284406 (PMC10146502; doi:10.1371/journal.pone.0284406)
Supplement: S4 Table — (PDF) [file pone.0284406.s005.pdf]

S5 Table - Illustrative quotes indicating Collective Action - the operational work that people do to enact the STAR pathway

Participant identifiers correspond to site and either Extended Scope Practitioner (ESP), or Consultant (COS).

"Relatively easily. I mean it would just require setting up a clinic template. They're just quite lengthy, so questions would be asked … So of course the management would look at that and they'd want some evidence that spending that length of time with the patient is effective. Cost effective and clinically effective." (Site 3/ESP1)

"I suppose time would be one thing in terms of the telephone follow-up calls with regards to the frequency of them ... I think it would have to be shown to be cost effective and beneficial in terms of the patient for that to be incorporated, for the STAR pathway to be incorporated in a higher degree, you know, into normal clinical practice." (Site 2/ESP1)

"So the funding is the main issue really … To have a specialist practitioner intervening and doing that and spending you know, a morning a week phoning up people you know, that's quite resource-heavy" (Site 2/COS2)

"Erm, disrupt – I don't think it disrupts anything. I think it just – you've just got to be careful haven't you, how you broach it because [yeah] I think – you know, you can't just ring and say 'I've seen this x-ray of this patient all over the place'. You've got to broach it in a very careful way and just say 'Would you mind looking at it?'." (Site 6/ESP1)

"[*Do you think that the staff in your organisation had a shared understanding of the purpose of the clinics and calls?*] That's a really good question. I think variable. I think that some people did. Obviously those of us working within it [yeah] did. I was never quite sure how on-board all the consultants were with it. You know, if I would write back to them, you're making sure you kind of do that in a sensitive and professional way." (Site 5/ESP1)

"Yeah, I don't know how it's regarded by erm – how it's seen amongst the surgeons. Whether it's interfering or…yeah. (Site 1/ESP2)

"I think some of them possibly think that we're looking to see whether there's something wrong from an orthopaedic point of view which then concerns them and puts them off a bit." (Site 4/ESP1)

"I think what's quite nice in [Hospital] is that actually, we're creating almost a team of people that are highly specialised in knee replacement follow-up. I think that's an excellent thing … for a patient, if they know everybody they're seeing on the pathway is highly specialised, knows all the answers about that procedure, it's gotta be an advantage for the patient." (Site 5/COS1)

"I think if anything it sort of helps to keep everybody in the loop really. Because I know from the assessment clinics, I know [other ESP] would always copy in the actual consultant that performed the surgery or the patient's care is under. And like I say, we routinely discuss […] patients that are struggling, with our own consultants […] So from that point of view I think it would help to improve those relations, rather than hinder them." (Site 2/COS1)

“I mean the training was excellent, yeah. I felt that – you know, for me in order to do this role, yeah, to attend the training was very clear, knew that I had access to erm the team if there were any questions.” (Site 5/ESP1)
