# Supplementary material for: Healthcare professionals’ views on implementing the STAR care pathway for people with chronic pain after total knee replacement: A qualitative study
Source: PLoS One. 2023 Apr 28;18(4):e0284406. doi: 10.1371/journal.pone.0284406 (PMC10146502; doi:10.1371/journal.pone.0284406)
Supplement: S5 Table — (PDF) [file pone.0284406.s006.pdf]

**S6 Table – Illustrative quotes indicating Reflexive Monitoring - The appraisal work that people do to understand how the STAR pathway affects them.** <br>**Participant identifiers correspond to site and either Extended Scope Practitioner (ESP), or Consultant (COS).**
"My clinical practice and my assessment and management of patients with knee arthritis and pain after knee replacement has changed because of all this. So I'm much more careful about the nature of pain that people have before surgery, I have a much lower threshold to identify and treat psychosocial issues and neuropathic pain." (Site 1/COS1) <br>"I think it's I guess confirmed some of the suspicions that I had about a certain group of patients post-TKR and how they do seem to be vulnerable, prone to developing neuropathic pain." (Site 3/ESP1) <br>"I mean I think it enhances patient care. For me it validates what we do anyway. I've really enjoyed the process of exploring patient expectation and satisfaction because I think that's where the current evidence base suggests we need to be focusing. Not just kind of the structure of the knee replacement and the biomedical side of things but thinking about the kind of biopsychosocial and the expectation management." (Site 5/ESP1) <br>"I'm not sure that I would modify it to be honest with you. Once we got to grips with it erm and once we organised it better, it all went pretty smoothly. Patients weren't always available when they said they would be for telephone reviews, which was a bit of a nuisance." (Site 3/ESP1) <br>"I think definitely you know, the telephone calls I think is the big thing. You've gotta try and find a way in which that's a little bit better, whether you get the patients to ring in at a certain time, whether you say to them, 'These are the appointments where the physio will be available. If you want to ring up and make an appointment to have a ten minute consultation', it might be the best way forward. Rather than the research assistants or the physio having to chase the patients up. It is time consuming." (Site 6/ESP1)
